# Familiarity Facilitates Detection of Angry Expressions

**DOI:** 10.3390/brainsci13030509

**Published:** 2023-03-18

**Authors:** Vassiki Chauhan, Matteo Visconti di Oleggio Castello, Morgan Taylor, Maria Ida Gobbini

**Affiliations:** 1 Department of Psychological and Brain Sciences, Dartmouth College, Hanover, NH 03755, USA; vchauhan@barnard.edu (V.C.);; 2 Department of Neuroscience and Behavior, Barnard College, Columbia University, New York, NY 10027, USA; 3 Helen Wills Neuroscience Institute, University of California, Berkeley, CA 94720, USA; 4 Department of Psychology and Neuroscience, Duke University, 417 Chapel Drive, Durham, NC 27708, USA; 5 IRCCS Istituto Delle Scienze Neurologiche di Bologna, 40139 Bologna, Italy; 6 Department of Medical and Surgical Sciences, University of Bologna, 40126 Bologna, Italy

**Keywords:** face perception, familiar faces, facial expressions, emotion, visual search

## Abstract

Personal familiarity facilitates rapid and optimized detection of faces. In this study, we investigated whether familiarity associated with faces can also facilitate the detection of facial expressions. Models of face processing propose that face identity and face expression detection are mediated by distinct pathways. We used a visual search paradigm to assess if facial expressions of emotion (anger and happiness) were detected more rapidly when produced by familiar as compared to unfamiliar faces. We found that participants detected an angry expression 11% more accurately and 135 ms faster when produced by familiar as compared to unfamiliar faces while happy expressions were detected with equivalent accuracies and at equivalent speeds for familiar and unfamiliar faces. These results suggest that detectors in the visual system dedicated to processing features of angry expressions are optimized for familiar faces.

## 1. Introduction

Faces provide a vast range of information about people that we rely on for social interactions [1,2,3,4,5,6,7]. Personal familiarity plays a critical role in tuning the face processing system [3,5,6,8,9]. In comparison to faces of strangers, familiar faces have a more robust representation [10,11] and can be detected more readily even with reduced attention and without conscious awareness [12].

According to a functional model on the distributed neural system for face perception [13] the encoding of structural aspects of a face for recognition of identity is performed mainly by the ventral visual pathway while processing of facial movements and, more generally, biological motion is performed by the dorsal visual pathway [3,14,15,16,17]. In previous work [18], we tested whether social cues, such as eye gaze direction and head angle, which are supposedly processed by the dorsal pathway, were detected more efficiently when conveyed by familiar faces. The results using a visual search paradigm showed that participants were faster to detect eye gaze direction and head position when conveyed by the face of a friend in comparison to a stranger. Therefore, familiarity of a face affects not only the visual representation of invariant aspects for identification, but also the perception of subtle changes that can signal an internal state, such as direction of attention. Here, we wanted to further investigate the detection of social cues. With this aim, we employed a visual search paradigm to measure the difference in detecting facial expressions of emotion when displayed by a personally familiar face and faces of strangers.

Facial expressions are efficient tools for nonverbal communication. Fine tuning to facial features, particularly in the ability to perceptually pick up on muscle activations, has been argued to be important for recognizing emotions [19]. A large body of the literature has investigated emotion recognition and capture of attention [20]. Prior studies have focused on which emotional expression has a greater advantage in capturing attention, and whether facial expressions of emotion are processed pre-attentively. Williams and colleagues (2005) [21] showed that both expressions of happiness and anger presented in an array of distractors (faces with neutral expression) are detected faster than other expressions such as sadness and fear. On the other hand, Calvo and Marrero (2009) [22] found that **happy expressions are detected faster than angry or sad expressions and concluded that** this difference in performance might be driven by low level features, such as the presence of teeth in happy expressions. The advantage for detecting faster happy expressions as compared to angry expressions in a visual search paradigm is further supported by Becker et al., (2011) [23], who found that happy faces are detected faster than angry faces, even when faces were computer generated and low-level feature differences were controlled. Relatedly, feature-based detection of emotional expressions has been established by the use of upright and inverted happy and angry expressions in a visual search paradigm, revealing better recognition performance in the former condition [24]. Manipulation of saliency of features, particularly around the mouth region, further reinforces the relationship between attention orientation and detection performance for different facial expressions in a visual search paradigm [25]. The literature on the contrast between happy and angry expressions in visual search paradigms, compounded with the reliability in recognition of these two particular emotional expressions (Kirouac & Doré, 1983) [26], motivated us to use these same expressions in our study. We were interested in exploring whether the detection of one or both types of expressions would be facilitated by personal familiarity.

The natural, repeated and extensive social experience with personally familiar faces leads to representations that optimize processing of these socially relevant stimuli. This optimization occurs at multiple levels in the distributed system for face perception: ***optimization of early visual processes*** that precede the activation of a view-invariant representation and explicit recognition of identity [12]; ***optimization of later visual processes*** with a view-invariant representation of personally familiar faces, which is dramatically more robust and efficient, affording effortless recognition of identity over large variations in image quality [27]; ***optimization of post-perceptual processes*** with spontaneous activation of person knowledge and an appropriate, individual-specific emotional response to personally familiar faces which might facilitate further efficient recognition through top-down processes [8]. In general, internal features might drive recognition of faces that a person has seen multiple times as opposed of those that have only been encountered once [28]. We hypothesized that one of the advantages for visual processing of familiar faces might involve the development of detectors for visual features that are specific to over-learned familiar faces. Activation of these detectors may facilitate further processing, such as driving saccades, attracting attention, or breaking through inter-ocular suppression, before the features are integrated into an explicit representation of the face that is view-invariant and linked to the face’s identity. Using a visual search task and familiar and unfamiliar faces as targets, presented either in an upright or inverted position, we have shown that familiar faces are detected around 100 ms faster in comparison to the faces of strangers. The optimized detection of identity might be, at least in part, supported by identity-related feature detectors in retinotopic visual cortex that are strengthened by familiarity [29].

The present experiment was motivated by the hypothesis that familiar face recognition exploits identity-specific local facial features. In a visual search paradigm where the target was a specific facial expression of emotion, we measured the accuracy and response time to two different emotional expressions displayed by familiar faces as well as faces of strangers. The results of this experiment provide further support to the hypothesis that the advantage of familiar face recognition relies, at least partially, on a feature-based type of processing. Moreover, results of this experiment contribute to the literature on the interplay between the ventral and dorsal pathways for recognition of identity [30].

## 2. Materials and Methods

### 2.1. Participants

A total of 15 graduate students (9 female, age: 25.6 ± 2.0) from Dartmouth College participated in the experiment. The sample size was chosen to be consistent with previous studies using the same paradigm [18,31,32]. All participants had normal or corrected to normal vision. Participants were recruited from the graduate students of the department of Psychological and Brain Sciences who were part of the program for at least one year. All participants provided written informed consent to participate in the experiment and were monetarily compensated for their time. The study was approved by the Dartmouth Committee for the Protection of Human Subjects (Protocol 29780).

### 2.2. Stimuli

The stimulus set consisted of 34 images. Of those, 24 images were of target identities, 12 of which were personally familiar to all of the participants (fellow graduate students), and 12 of which were unfamiliar controls, chosen to be visually similar to the familiar identities. A total of 10 images were of distractor identities (5 male and 5 female). To ensure that the graduate students were not visually familiar with the unfamiliar targets and the distractors, images of unfamiliar identities were collected at the Massachusetts Institute of Technology. Three images per target identity were used—one image for angry, happy, and neutral expressions each. In this experiment, we used 4 familiar identities (2 male and 2 female) and 4 unfamiliar identities (2 male and 2 female) matched for gender, ethnicity and age to the familiar targets. All stimuli were collected with the same camera, placement, settings, and lighting equipment to minimize differences due to image quality. They were cropped to be the same size (350 × 350 pixels) using custom code written in Matlab. To further reduce low level visual differences, the average pixel intensity of each image (ranging from 0 to 255) was set to 128 using the SHINE toolbox function lumMatch in Matlab [33].

The stimuli were acquired under controlled standardized parameters in the laboratory, with the same camera and lights placed at the same distance from the stimulus model across all individuals who consented to being photographed and having their images used for research in accordance with the Dartmouth Committee for the Protection of Human Subjects (Protocol 29780). In order to provide visual prompts, we showed stimulus model images of facial expressions from the NimStim facial expression database [34]. Models were asked to maintain a relaxed resting expression and then make the facial expression they were prompted to perform by the experimenter. The collected images were placed in the center of a black background, cropping the shoulders but retaining portions of the neck and hair. We did not acquire ratings for these stimuli from an independent group of subjects, but all the participants of the current study provided ratings for how expressive the facial expression was for all the target faces after the experimental session.

**Figure 1:**
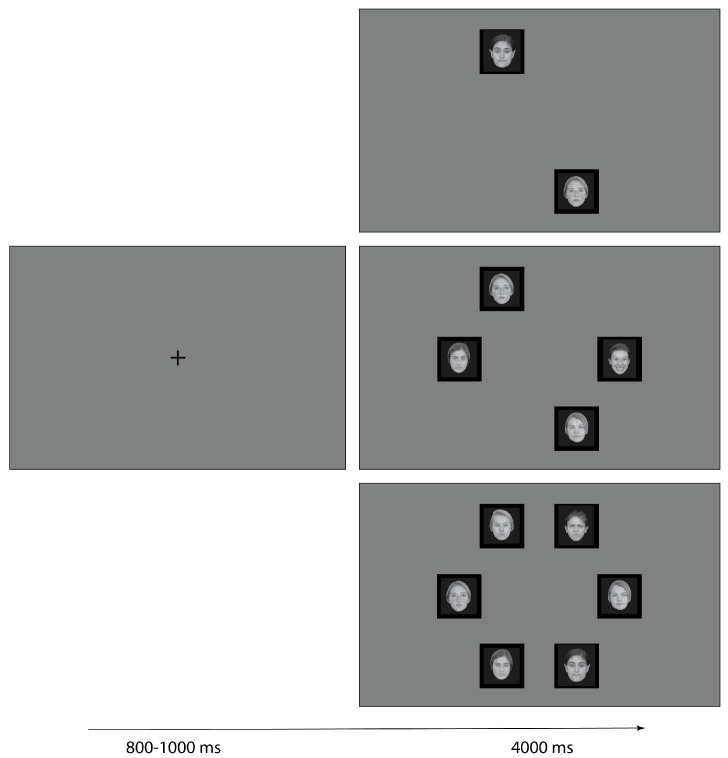
Example sequence of events in a single trial. The fixation cross was on the screen for a jittered interval between 800 and 1000 ms. The array of face stimuli stayed on screen until the participant responded, or for a maximum of 4 s. The subsequent trial began immediately after the participant’s response. Stimulus arrays were sets of 2, 4, or 6 face images. Example stimulus arrays for target absent trial (**top**), target present trial in the happy condition (**middle**), and target present trial in the angry condition (**bottom**) are depicted in the right panel of the figure. The happy and angry conditions were blocked.

During the task, stimuli were presented in sets of two, four, or six. These images were presented symmetrically, placed in a regular hexagon, centered on the fixation cross, such that the center of each image was always at 7° of visual angle from the fixation cross (Figure 1). Each image subtended 4° × 4° of visual angle. The positions of the images were chosen to be symmetric around the fixation for each set size.

### 2.3. Experimental Setup

The experiment was run on a GNU/Linux workstation with presentation scripts written in MATLAB (R2014b) using Psychtoolbox (version 3.0.12). The resolution of the screen was 1440 × 900 pixels and the refresh rate was 60 Hz. Participants sat approximately 50 cm from the screen with their face resting on a chin rest. All stimuli were presented against a uniform gray background.

### 2.4. Task

The first phase of the experiment involved familiarization with the target stimuli. The participants saw each image of familiar and unfamiliar targets making angry and happy expressions twice, for 4 s each time, with a 500 ms inter-stimulus interval between each image.

Following the familiarization phase, participants performed 10 practice trials identical in structure to the experiment (the practice trials were not included in the final analysis). After the practice trials, the experiment started, and participants performed 2 blocks of 192 trials each. In one block, the target was a face with an angry expression among neutral distractors. In the other block, the target was a face with a happy expression among neutral distractors. The identity of the target stimulus with an angry or happy expression was familiar in half of the trials and unfamiliar in the other half. On target-present trials, the identity of distractor faces with neutral expressions were always unfamiliar. On target-absent trials, one face with a neutral expression was a familiar identity in half of the trials. The order of blocks was counterbalanced across participants. Participants were asked to press the left arrow key if the target was present and the right arrow key if the target was absent. They had a maximum of 4 s to make their response. Participants were instructed to respond only to the expression of the target and to ignore the identity of the target. Participants were also instructed to respond as quickly as possible, but not at the expense of accuracy.

Each block began with the instructions specifying which expression was the target for the following trials (e.g., “look for happy expression”). Each trial ended when the participant’s response was recorded or ended after a maximum of 4 s. The inter-trial interval was jittered between 800 milliseconds and 1 s. Participants fixated a central fixation cross during the inter-trial interval, and the cross disappeared once the face stimuli appeared, allowing eye movements to the stimuli. Every 48 trials, the participant was given the option of taking a short break. For each block, each unique trial type was repeated 4 times. A trial type is defined by a given set size (2, 4, or 6), target condition (present or absent), and the unique identity of the target (8 identities—4 familiar, 4 unfamiliar). The sex of the distractors always matched the sex of the target. Targets with the emotional expression were presented in half the trials for each block. Targets were equally presented in the left and right hemi-field.

After the visual search session was over, participants rated the target stimuli on how recognizable the happy and angry expressions were. In order to rate the expressions of the targets, participants judged how clearly the face expressed the emotion of anger or happiness by choosing a number between 1 and 5 (with 5 being maximally expressive).

### 2.5. Statistical Analyses

Accuracies were analyzed using the function *`glmer’* from the package *`lme4’* [35] and the function *`Anova’* from the package *`car’* [36]. The analysis of accuracy involved fitting a generalized linear mixed model to the data—with accuracy as the dependent variable; target presence, set size, target expression, target familiarity, and the interactions between these variables as fixed effects; the participant and stimulus identities as random effects. We also included the sex of the target as a fixed effect in our model. We compared models with different random slopes and intercepts until we identified the best model with the lowest Akaike’s information criteria (AIC). To identify which fixed effects significantly affected the accuracy, we used Type 3 analysis of deviance (using Wald’s $\chi^{2}$ test).

For the analysis of reaction times, we used the function *`lmer’* from the package *`lme4’* [35] and the function *`Anova’* from the package *`car’* [36]. We fitted our data with a logit mixed model with log-transformed reaction times as the dependent variable and target presence, set size, target expression, target familiarity, and the interactions between these variables as fixed effects. We also added the sex of the target as a fixed effect. The random effects included participants and stimulus identities. The model complexity was reduced by removing random slopes and intercepts until the best model was identified using the lowest AIC value. Again, we used Type 3 analysis to determine the significance of the fixed effects entered in the model. The bootstrapped confidence intervals for the figures and the effect sizes were estimated with custom code written in R. The 95% confidence intervals were calculated by randomly sampling subsets of trials from each participant across conditions to compute the dependent variable (accuracy, reaction time, and rating). These values are reported within square brackets in the results. For both models, we used a polynomial contrast for set size and zero sum contrasts for the remaining fixed effects.

## 3. Results

### 3.1. Accuracy

**Figure 2:**
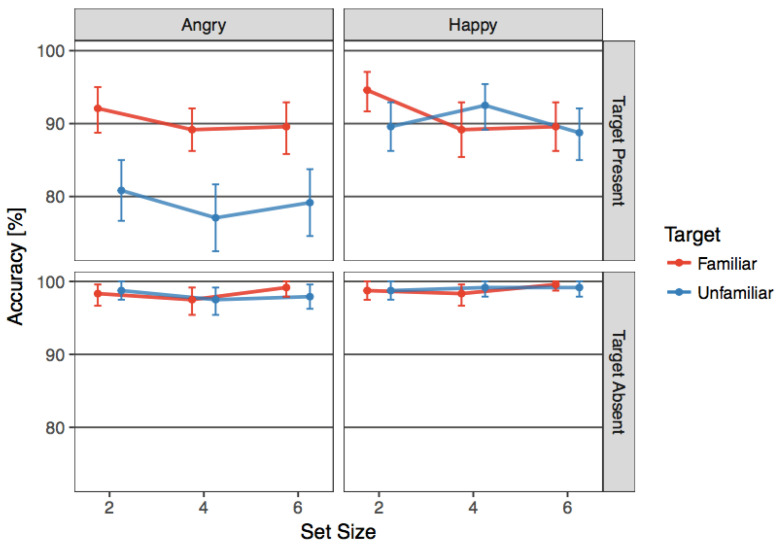
Accuracy in reporting presence or absence of target expression as a function of set size of images. Rows depict target present or absent condition and columns depict the target expression for the block. The effect of familiarity on accuracy is driven by greater accuracy for familiar faces when the target is present, and the target expression is angry. Error bars indicate 95% bootstrapped confidence intervals.

We fitted generalized linear mixed models to our data, with accuracy as the dependent variable. We used two separate models to analyze the accuracy for the target present and absent conditions, consistent with prior literature (Tong & Nakayama, 1999). For the target absent trials, participants performed at nearly 100% accuracy across all conditions (Figure 2).

In the target present condition, the fixed effects in the model with the lowest AIC were set size, familiarity of the target, target expression, and their interactions. We also added the fixed effect of target sex. The random effects for this model included slopes and intercepts for participants. The set size of the items in the search array did not have a significant effect on the accuracy ($\chi^{2}$(2) = 4.74, *p* = 0.09). We found significant effects of familiarity ($\chi^{2}$(1) = 21.03, *p* < 0.001) and expression ($\chi^{2}$(1) = 21.43, *p* < 0.001). The interaction between these two fixed effects was also found to be significant ($\chi^{2}$(1) = 12.15, *p* < 0.001) while none of the other interactions were found to be significant (set size × familiarity: ($\chi^{2}$(2) = 4.41, *p* = 0.1), set size × expression: ($\chi^{2}$(2) = 1.16, *p* = 0.55), set size × familiarity × expression: ($\chi^{2}$(2) = 3.0, *p* = 0.22)). Lastly, we also found a significant effect of the sex of the target ($\chi^{2}$(1) = 4.02, *p* = 0.04) (Figure 2). The analysis of individual contrasts revealed that subjects were more accurate for familiar targets overall (familiar: 94.65% [93.96,95.35], unfamiliar: 91.60% [90.73, 92.43]; numbers in brackets indicate the bootstrapped 95% confidence intervals), and for targets with a happy expression as compared to the angry conditions (happy: 94.83% [94.10, 95.56], angry: 91.42% [90.56, 92.29]). The significant interaction between the two terms reflected that the advantage of familiarity on accuracy in the target present condition was found only for angry targets (90.28% [88.33, 92.08] versus 79.03% [76.53, 81.53]) and was absent for happy targets (91.11% [89.17, 92.92] vs. 90.28% [88.33, 92.22]).

### 3.2. Reaction Times

**Figure 3:**
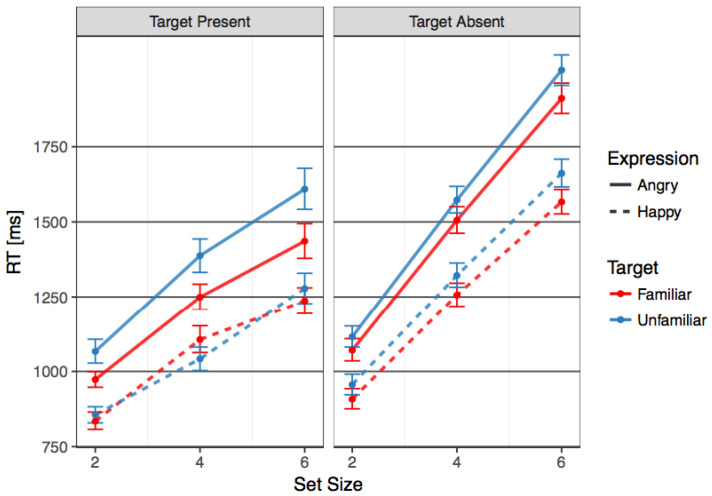
Reaction times (ms) as a function of set size of images. Longer reaction times were observed for unfamiliar faces when the target was an angry expression. Error bars indicate 95% bootstrapped confidence intervals.

Only correct trials were included in the analysis of reaction times. We fitted a linear mixed model to our data, with log-normalized reaction times as our dependent variable. As with accuracy, we analyzed reaction times in the target present and absent conditions separately. The main effects in the model with the lowest AIC included set size, familiarity of the target, target expression, and the interactions between these variables. We also included the main effect of target sex in the model. The random effects included slopes and intercepts for the participants and the slopes for the different image combinations. We found a significant effect of set size ($\chi^{2}$(2) = 504.77, *p* < 0.001), target familiarity ($\chi^{2}$(1) = 11.13, *p* < 0.001), target expression ($\chi^{2}$(1) = 206.64, *p* < 0.001), and target sex ($\chi^{2}$(1) = 37.04, *p* < 0.001). The interaction between target familiarity and expression was also found to be significant ($\chi^{2}$(1) = 12.47, *p* < 0.001), while none of the other interaction terms were significant (set size × familiarity: ($\chi^{2}$(2) = 3.26, *p* = 0.19), set size × expression: ($\chi^{2}$(2) = 2.29, *p* = 0.31), set size × familiarity × expression: ($\chi^{2}$(2) = 2.96, *p* = 0.22). Overall, in the target present condition, familiar targets were detected 135 ms [95, 175] faster than the unfamiliar targets for angry expressions, and 1 ms [−33, 31] slower for happy expressions (Figure 3).

**Figure 4:**
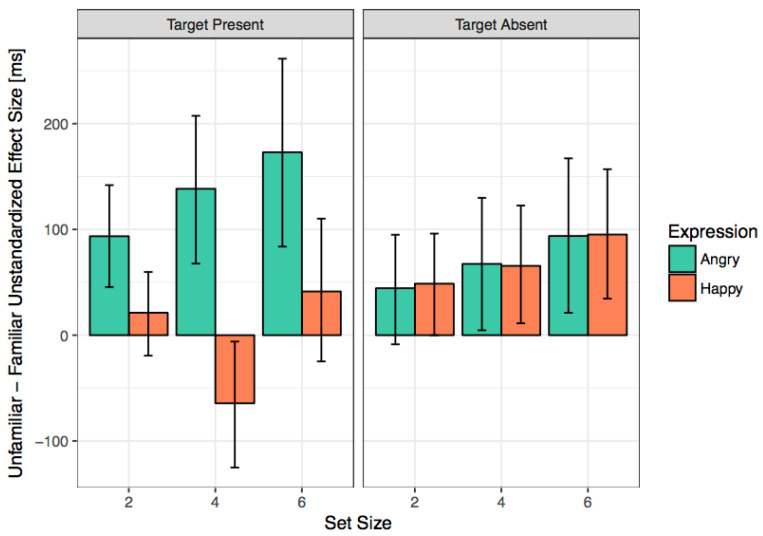
Unstandardized effect sizes for the difference in reaction times between unfamiliar and familiar faces as a function of set size. Error bars indicate 95% bootstrapped confidence intervals.

In the target absent condition, the dependent variable was log-transformed reaction time and the main effects were set size, target familiarity, target expression, and the interactions between these terms. Target sex was also included as a fixed effect. The random effects included random slopes and intercepts for participants and random slopes for different image combinations. We found significant main effects of set size ($\chi^{2}$(2) = 1964, *p* < 0.001), target familiarity ($\chi^{2}$(1) = 18.71, *p* < 0.001), target expression ($\chi^{2}$(1) = 449.53, *p* < 0.001), and target sex($\chi^{2}$(1) = 4.67, *p* = 0.03). None of the interactions were found to be significant (familiarity × expression: ($\chi^{2}$(2) = 0.07, *p* = 0.78), set size × familiarity: ($\chi^{2}$(2) = 0.29, *p* = 0.86), set size × expression: ($\chi^{2}$(2) = 3.46, *p* = 0.17), set size × familiarity × expression: ($\chi^{2}$(2) = 0.0, *p* = 0.99). Interestingly, despite the absence of a target making an expression, we found that subjects were faster in making a response when the task was to report the presence of a target with a happy expression (1.28 s [1.26, 1.29]) as compared to the angry expression (1.53 s [1.51, 1.55]) (Figure 3). Moreover, even though no targets with expressions were present in the stimulus array—either a familiar or an unfamiliar identity with a neutral expression was included as a distractor—it was, therefore, possible for us to analyze the effect of the presence of a familiar distractor in the search array even in the trials when there was no target with an emotional expression. We found that participants responded that a target was absent faster when a familiar face with a neutral expression was among the stimuli, as compared to target absent trials with all unfamiliar faces. This difference was seen both in angry target and happy target trials (69 ms [33, 105] and 70 ms [38, 101] differences for angry target and happy target trials, respectively). Unstandardized effects for reaction times in both target present and absent conditions are depicted in Figure 4.

### 3.3. Expression Ratings

We also analyzed the ratings for how recognizable happy and angry expressions were in each target identity. We fitted a generalized linear mixed model to the rating data, with the rating for how recognizable the expression was as the dependent variable, and target familiarity, expression, and sex as independent variables. The model with the lowest AIC included subject specific slopes and intercepts as random effects. We used the Poisson distribution as the linking function for this model.

**Figure 5:**
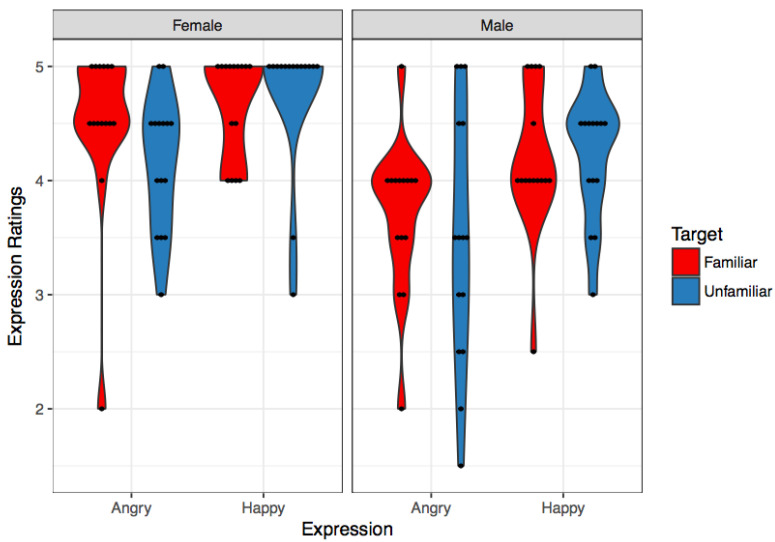
Ratings for recognizability of expression (1: not expressive, 5: very expressive) as a function of target expression. Left panel is for female targets, right panel is for male targets. Ratings were lower for male targets as compared to female targets and angry expression compared to happy expression. Error bars indicate 95% bootstrapped confidence intervals. Black dots represent responses from individual subjects.

We found a significant effect of target sex on the ratings ($\chi^{2}$(1) = 5.52, *p* = 0.02), which was driven by lower recognizability ratings for the males (3.91 [3.83, 3.99]) as compared to the females (4.53 [4.46, 4.59]). We did not find a significant effect of the target expression ($\chi^{2}$(1) = 3.58, *p* = 0.06), or of target familiarity ($\chi^{2}$(1) = 0.172, *p* = 0.68). The interaction between target familiarity and expression was not significant ($\chi^{2}$(1) = 0.43, *p* = 0.51), nor was the trend for ratings of familiar versus unfamiliar angry expressions ($\chi^{2}$(1) = 0.53, *p* = 0.46) (Figure 5).

## 4. Discussion

In this study, we investigated whether personal familiarity facilitates the detection of emotional facial expressions. The results provided support for our hypothesis (faster detection of expressions of emotion when displayed by familiar faces) for detection of angry facial expression but not, interestingly, for happy expressions. We found that participants were more accurate and faster at detecting an angry facial expression if the face making that expression was personally familiar. The effect was large, with an 11% difference in detection accuracies and a 135 ms advantage in detection time. After the visual search task, participants were asked to rate the expressions. Angry expressions of unfamiliar faces were rated as being equally expressive as the angry expressions of personally familiar faces, indicating that the accuracy and speed differences in the visual search task were not due to differences in expression ambiguity.

Previous research has shown that familiar identities are detected faster, as compared to unfamiliar identities [12,32,37,38]. Moreover, the effect of familiarity on detecting familiar identities is robust when face images are inverted [32], suggesting that familiarity-based facilitation can also involve parts-based, rather than holistic, face perception processes. Social cues, such as head angle and eye gaze, also are detected faster when conveyed by familiar faces, as compared to unfamiliar faces [18], with a speed advantage similar to that found in the current study for detection of angry facial expressions.

The current study shows that familiarity-based facilitation of social cue detection extends to emotional expressions, which are conveyed by contractions of facial muscles [39,40]. Thus, the facilitation of visual processing of faces that accrues from learning socially-salient, personally familiar faces involves both the detection of invariant features that specify identity and the detection of changeable features that convey facial gestures and social signals—processes that are mediated by the ventral and dorsal pathways, respectively, in the core system of the human neural system for face perception [13,41,42]. We have further shown that visual learning of familiar faces affects retinotopic biases for identity recognition, suggesting that this visual learning extends to early processes in the retinotopic visual cortex [29]. This body of work is consistent with our hypothesis that visual learning of familiar faces involves the development of detectors for individual-specific fragments of a familiar face that facilitate detection of that face’s identity and gesture.

Familiar face perception also spontaneously evokes representations of person knowledge—that person’s dispositions, personality, and position in a social network [3,4,5,8,9,42,43,44]. The effect of person knowledge on detection of facial expression, which may be mediated by a top-down mechanism, may also play a role in familiarity-based facilitation.

Interestingly, familiarity-based facilitation did not extend to detection of happy expressions. Participants detected happy expressions conveyed by familiar and unfamiliar faces with equivalent accuracy and at equivalent speeds. Our stimuli with happy expressions were detected faster and more accurately than our stimuli with angry expressions. In general, others have found that happy expressions are recognized with the highest accuracy compared to the other canonical expressions [26,45,46,47]. In experiments using a visual search paradigm with facial expressions conveyed by unfamiliar faces, an advantage for happy expression in comparison to other facial expressions of emotion such as anger, sadness, fear, disgust and surprise has been reported [21,22,48]. The absence of an effect of familiarity on the detection of the happy expression suggests that this process is optimized, perhaps because it is such a common social cue for interactions with both familiar and unfamiliar others. This effect may also be explained by low-level features that distinguish happy and neutral expressions such as exposed teeth, even though we did not observe a pop out effect in the visual search task [49]. Moreover, work by Becker and colleagues [23] demonstrates that happy expressions are detected faster than angry expressions in a visual search task even when the stimuli conveying the happy expression do not exhibit exposed teeth. Our finding of faster detection for the happy expression, as compared to the angry expression, is consistent with several other experiments that happy expressions are detected more efficiently compared to other emotional expressions such as anger, sadness, fear, disgust, and surprise [21,48]. Our results show that familiarity does not affect detection of happy expressions since no significant difference was recorded for familiar and unfamiliar targets. Further research could determine if this familiarity invariance extends to subtler, unposed, or less stereotypic expressions of happiness. Similarly, further research could determine whether familiarity-based facilitation is also found for other standard expressions, such as disgust and surprise, as well as for non-standard expressions, such as contempt, boredom, and skepticism.

We found an unexpected effect of familiarity on target absent trials. Faster responses on target absent trials in which one stimulus was a familiar face with a neutral expression suggests that participants adopted a strategy in which they may have terminated search when they detected this familiar distractor, forgoing examination of the other unfamiliar distractors. This suggests that the participants were using the strategy of performing a self-terminating visual search [50], and relying on the familiar face as an indicator of whether an emotional expression target was present or absent.

The face perception system plays a central role in social interactions and mounting evidence shows that it is optimized for interactions with personally familiar others. This optimization is evident in both detection of invariant facial features that specify identity, and changeable facial features that carry social signals. Optimization involves visual learning, based on extended naturalistic interactions with personally familiar others, and involves both the ventral and dorsal face pathways in the distributed system for face perception and extends to early processes in retinotopic cortex. Familiar face perception, unlike unfamiliar face perception, also involves the spontaneous activation of neural systems for the retrieval of person knowledge. Understanding face perception—its perceptual and cognitive processes, its neural substrates—requires understanding how it processes familiar faces, in much the same way that understanding language processing and its neural substrates requires understanding how one processes one’s native language.

In addition to providing further support to the hypothesis that familiar faces are processed in an optimized way, presumably as a result of an advantage in feature-based processing, our results contribute to the literature on separate pathways for processing identity and emotion. Evidently, we can recognize a facial expression even if we don’t know the identity of the person displaying that emotion. An early cognitive face model posited that identity and emotion are processed separately (Bruce and Young, 1986). However, recognition of an expression can clearly be aided by repeated visual familiarization with the identity of the face that signals that expression. Faster detection of angry expression conveyed by familiar faces suggests an interplay between emotion and identity recognition [30,51,52].

## Data Availability

Code and data for the study can be obtained by contacting the corresponding author.
